# Minimal access median sternotomy for aortic valve replacement in elderly patients

**DOI:** 10.1186/1749-8090-8-103

**Published:** 2013-04-20

**Authors:** Yousuf Alassar, Yalin Yildirim, Simon Pecha, Christian Detter, Tobias Deuse, Hermann Reichenspurner

**Affiliations:** 1 Department of Cardiovascular Surgery, Univeity Heart Center Hamburg, Martinistr. 52, Hamburg, 20246, Germany

**Keywords:** Aortic valve replacement, Minimal Access Sternotomy, Limited skin incision

## Abstract

**Background:**

We report our clinical experience with a approach for aortic valve replacement (AVR) via minimal access skin incision and complete median sternotomy. This approach was used in patients with higher age and multiple co-morbidities, facilitating an easy access with short bypass and cross clamp times. It was especially performed in patients asking for an excellent cosmetic result, who did not qualifying for minimally-invasive AVR via partial upper sternotomy.

**Methods:**

AVR via minimal-access median sternotomy, was performed in 58 patients between 01/2009 and 11/2011. Intra- and postoperative data including cross clamp time, cardiopulmonary bypass time, mortality, stroke, pacemaker implantation, re-operation for bleeding, ventilation time, ICU and hospital stay, wound infection, sternal dehiscence or fracture and 30 day mortality were collected.

**Results:**

Mean patients age was 76.1 +/−9.4 years, 72% were female. Minimal-access AVR could be performed with a mean length of midline skin incision of 7.8 cm. Aortic cross-clamping time was 54.6 +/−6.3 min, cardiopulmonary bypass time 71.2+/−11.3 min and time of surgery 154.1 +/−26.8 min. Re-operation for bleeding had to be performed in 1 case (1.7%). There were no strokes or pacemaker implantations needed. Mean ventilation time was 4.5 h, ICU stay was 2 days and mean length of hospital stay was 6 days. 6 months follow up showed mortality of 0% and no sternal dehiscence or wound infection was observed.

**Conclusion:**

Minimal-access AVR via complete median sternotomy can be performed safely,in this elderly patient cohort without adding additional operative risk compared to conventional AVR. By avoidiance of large skin incisions this approach combines excellent cosmetic results with fast surgery time and excellent postoperative recovery.

## Background

Conventional aortic valve replacement (AVR) via complete median sternotomy is a safe and feasible procedure with low risk for patients providing excellent long-term outcome [1-3]. Over the last two decades, different minimally-invasive approaches for AVR have been developed and are increasingly being utilized. There are different approaches described, such as partial upper hemisternotomy, right parasternal thoracotomy or transverse sternotomy [4]. All these approaches have the aim of decreased invasiveness and less surgical trauma. Advantages of minimal invasive AVR have been shown as less postoperative pain, shorter ICU and hospital stays, shorter ventilation time, decreased blood loss and better cosmetic results with mortality and morbidity comparable to conventional sternotomy [1-4].

In patients with multiple co-morbidities minimally-invasive approaches may increase the overall operative risk, because longer cross clamp-, bypass- and surgery times compared to conventional AVR have been described. Because these patients may still ask for surgery with favorable cosmetic outcome, we offer minimal-access AVR which combines a limited skin incision (mean 7.8 cm, range 7.0-8.5 cm) with the surgical advantages and the safety (clear arrangement and easy access to the operation field, shorter operation time) of full sternotomy. We here report our cumulative experience with this modified approach.

## Methods

Between 01/2009 and 11/2011, minimal-access AVR through a full sternotomy was performed in 58 patients. Mean patient age was 76.1 +/−9.4 years, 72.0% were female. Mean Society of Thoracic Surgeons Predicted Risk of Mortality (STS-PROM) was 5.71%. Patient characteristics are shown in Table 1. A retrospective single-center data analysis was performed. Intra- and postoperative data were collected. Three months follow up including echocardiography and examination of the sternal stability by inspection and palpation was performed in our department in all patients.

**Table 1:** Patient characteristics

	**Patients n = 58**
Age (years)	76.1 ± 9.4
Gender (female/male)	42/16
Height (cm)	168 ± 6.8
Weight (kg)	73 ± 4.5
Body mass index	26 ± 3.4
Hypertension (n)	46
Diabetes (n)	23
Renal insuffiency (n)	16
COPD (n)	19
Cerebrovascular disease (n)	6
Peripheral vascular disease (n)	15
Coronary artery disease (n)	19
Previous MI (n)	7
Ejection fraction (%)	48.6 ± 9.3
Aortic stenosis (n)	53
Aortic insufficiency (n)	12
Combined vitium (n)	7
Endocarditis (n)	5
STS-PROM (%)	5.71

COPD Chronic obstructive pulmonary disease; MI Myocardial Infarction; STS-PROM Society of Thoracic Surgeons Predicted Risk of Mortality.

### Surgical technique

**Figure 1:**
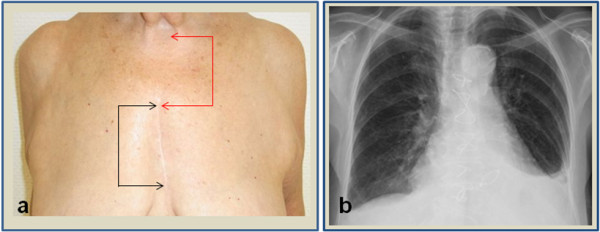
**Position of the scar.** (**a**) Scar (black arrows) position approximately 5 cm below jugulum (red arrows). (**b**) chest x-ray showing sclerotic aorta and adaptation of the sternum with standard steel wires.

**Figure 2:**
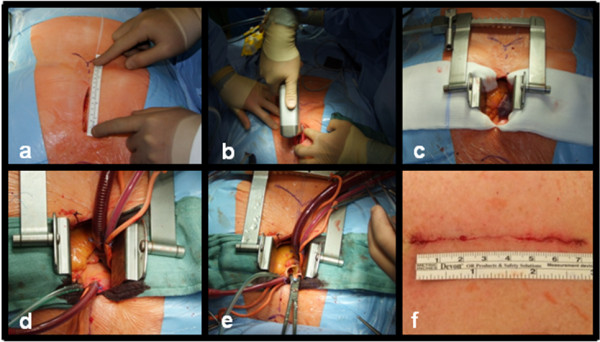
**Intraoperative situs.** (**a**) Limited skin incision is placed approximately 5 cm below jugulum. (**b**) A complete median sternotomy is performed using a pendulum saw. (**c**) Excellent access to the right atrial appendage and aortic root. (**d**) Clear arrangement and easy access to the operation field facilitates fast and save cannulation. (**e**) Aortic cross clamping and standard transverse aortotomy is conducted for AVR. (**f**) Intracutaneous suture of 7.5 cm.

A midline skin incision is started approximately 5 cm below the jugulum and extended to a maximum of 8.5 cm Figure 1a. The soft tissue over the body- and manubrium sterni is undermined to expose the xiphoid process and the suprasternal notch. A complete median sternotomy is performed using a pendulum saw Figure 2a, b. A retractor is inserted and the pericardium is opened through a vertical incision followed by traction sutures to expose the ascending aorta and the right atrial appendage. Two purse-string sutures are placed in the distal ascending aorta, another on the right atrial appendage. Aortic cannulation is performed using a 22 F (French). arterial cannula. For venous cannulation a 29/37 F. two-stage venous cannula is utilized. Aortic vent is placed on the ascending aorta, LV-Vent is inserted via right pulmonary vein Figure 2c, d. Cardiopulmonary bypass is started with mild systemic hypothermia (32°C) and cardiac arrest is induced by antegrade cold crystalloid cardioplegia (Custodiol HTK). After aortic cross clamping standard transverse aortotomy is conducted Figure 2e. Then aortic valve replacement is performed as it would be in case of a standard median sternotomy with conventional skin incision. At the end of the procedure the sternum is closed with 6 to 8 steel wires depending on the length of the sternum. Soft tissue is closed with absorbable suture Figure 2f.

### Statistical analysis

All statistical analysis was performed by JMP 9 Software (SAS Institute Inc, Cary, USA). Continuous values are expressed as mean ± standard deviation and categorical variables are displayed as percentages.

## Results

Intra- and postoperative data are shown in Tables 2 and 3. AVR could be performed successfully in all patients with a mean length of midline skin incision of 7.8 cm (range 7.0-8.5 cm). Mean BMI was 26.0 (range 20.0-32), but this approach could also be used successfully in five patients with BMI > 30. Aortic cross-clamping time was 54.6 +/−6.3 min, cardiopulmonary bypass time 71.2+/−11.3 min and time of surgery 154.1 +/−26.8 min. In 57 patients a bioprosthesis was used, one patient received a mechanical valve. Also implantation of stentless valves could be performed successfully via this access (n = 8). Mean size of implanted valves was 23 mm (21 mm-27 mm). All patients could be weaned from cardiopulmonary bypass without the need for postoperative mechanical circulatory support. One patient had to be re-operated for bleeding from the aortotomy site. This could be conducted without expansion of the skin incision. Mean ventilation time was 274 ± 143 min, mean ICU stay 1.9 ± 0.9 days and mean length of hospital stay was 6.0 ± 1.2 days. Intra- and postoperative Echocardiography showed good results in all patients. In five cases a minimal paravalvular leckage was ascertained, in all other patients prosthetic function was without insufficiency. Mean Gradient was 17/8 mmHg (max/mean). No reoperation due to prosthetic endocarditis or paravalvular leakage had to be done during early follow up. 30 day mortality was 0% and no stroke occurred. No pacemaker implantation was needed and no sternal dehiscence or wound infection occurred within 6 months follow up.

**Table 2:** Intraoperative data

	**Patients n = 58**
Aortic cross clamp time (min)	54.6 ± 6.3
CBP time (min)	71.2 ± 11.3
Time of surgery (min)	154.1 ± 26.8
Stented bioprosthesis (n)	49
Stentless bioprosthesis (n)	8
Mechanical prosthesis (n)	1
Mean valve size (mm)	23

**Table 3:** Postoperative data

	**Patients n = 58**
Mechanical ventilation time (min)	274 ± 143
ICU stay (d)	1.9 ± 0.9
Hospital stay (d)	6.0 ± 1.2
Reoperation for bleeding (n)	1
Pacemaker implantation (n)	0
30 day mortality (n)	0
Sternal wound infection (n)	0
Prosthetic valve endocarditis (n)	0

## Discussion

Over the last two decades several minimally invasive approaches to aortic valve surgery, such as partial upper hemisternotomy, right parasternal thoracotomy or transverse sternotomy have been increasingly used. In the majority of cases reported in literature the partial upper sternotomy has been utilized, showing some benefits of this minimally invasive approach compared to conventional sternotomy. The advantages have been shown as decreased blood loss, less postoperative pain, shorter length of hospital- and ICU stay, decreased ventilation time and better cosmetic results with comparable results regarding morbidity and mortality [5-8]. However in randomized controlled trials as well as meta-analysis by Murtuza et al. for mortality no statistically significant differences has been seen between patients receiving conventional- or minimally invasive AVR [2,7-9].

In our Institution, the partial upper hemisternotomy is the access of choice for minimally-invasive AVR and can be performed safely and feasible. However, there are some patients not qualifying for this approach due to different reasons. One group are older patients with many co-morbidities and the need for fast operation time. Most of the studies concerning minimally invasive AVR have reported longer cross clamp and cardiopulmonary bypass time as well as longer time of surgery compared to conventional AVR [4,6,10,11]. The median sternotomy facilitates symmetric retraction of the sternum, resulting in clear arrangement and easy access to the operation field. This is particularly important in patients with sclerotic Aorta (Figure 1b) where cannulation, cross-clamping and suturing of the aortotomy can be technically difficult. Furthermore implantation of stentless valves can be performed without problems. In our group of patients the new access by limited skin incision and full median sternotomy provided fast cross-clamp, cardiopulmonary bypass and surgery time, almost comparable to results published for conventional AVR without limited skin incision [6,9,12]. This is certainly enabled by the clear arrangement of the operation field after median sternotomy which allows easy access to the operation site. So these patients can probably benefit from the fast procedure time without giving up the excellent cosmetic result.

Especially in women, the cosmetic result of the operation is often very important. The skin incision with our approach is with mean length of 7.8 cm comparable to minimally invasive AVR by partial upper hemisternotomy and the shortest skin incision reported for complete sternotomy in literature [1,3,5,6]. The advantage of our access compared to a minimally invasive approach by partial upper sternotomy is the position of the scar. The beginning of our incision is lower (approximately 5 cm below the jugulum) which means that the upper part of the scar is covered by clothes, which can be important especially in women (Figure 1a).

In minimally invasive approaches without complete sternotomy a better chest wall stability compared to conventional AVR is reported [2,13]. This advantage cannot be reached with our approach, although no case of sternal dehiscence has been seen in our patients. Particularly in patients with multiple co-morbidities an intraoperative conversion to conventional approach can be necessary due to bleeding or technical difficulties. With our access this conversion is very easy and only needs an expansion of the skin incision which can be performed faster compared to other minimally invasive approaches where an expansion of the sternotomy needs to be done. In these cases with partial upper- and complete sternotomy this complex sternal fracture can be difficult to stabilize and is probably associated with higher potential for sternal dehiscence.

In patients with minimal invasive AVR via partial upper sternotomy, due to limited space, sometimes, venous cannulation via vena femoralis can be needed. Here the risk of groin complications is given [10]. This can be avoided by our approach due to the possibility of venous cannulation via right atrium in all cases based on excellent access to the right atrial appendage (Figure 2c).

In our cohort of patients 6-months follow up showed mortality of 0%, there was no need for permanent pacemaker implantation, no sternal wound infection and no stroke occurred. One revision due to bleeding had to be done. This could be performed without expansion of the skin incision. These results are very pleasant but regarding the examined number of patients comparable to other studies of minimally invasive AVR reported in literature [12,13].

## Conclusion

In this group of patients AVR by minimal-access and complete median sternotomy can be performed safely, combining excellent cosmetic results with short time of surgery and postoperative recovery.

## Abbreviations

AVR: Aortic valve replacement; ICU: Intensive care unit; STS-PROM: Society of Thoracic Surgeons Predicted Risk of Mortality; F: French; LV: Left ventricular; COPD: Chronic obstructive pulmonary disease; MI: Myocardial Infarction; BMI: Body mass index; CBP: Cardiopulmonary Bypass.

## Competing interests

The authors declare that they have no competing interests.

## Authors’ contributions

YA performed the surgery, contributed to the writing of the manuscript and the development of the study design. YY contributed to the writing of the manuscript and the development of the study design and performed data acquisition. SP contributed to writing of the manuscript and performed data acquisition and statistical analysis. CD performed the surgery and reviewed the manuscript. TD performed the surgery and contributed to the writing of the manuscript. HR performed the surgery and reviewed the manuscript. All authors read and approved the final manuscript.

## References

[B1] Von Segesser LK Westaby S Pomar J Loisance D Groscurth P Turina M Less invasive aortic valve surgery: rationale and technique Eur J Cardiothorac Surg 1999 15 6 781 785 10.1016/S1010-7940(99)00119-0 10431859

[B2] Bonacchi M Prifti E Giunti G Frati G Sani G Does ministernotomy improve postoperative outcome in aortic valve operation? A prospective randomized study Ann Thorac Surg 2002 73 2 460 465 10.1016/S0003-4975(01)03402-6 11845860

[B3] Tabata M Umakanthan R Cohn LH Bolman RM 3rd Shekar PS Chen FY Couper GS Aranki SF Early and late outcomes of 1000 minimally invasive aortic valve operations Eur J Cardiothorac Surg 2008 33 4 537 541 10.1016/j.ejcts.2007.12.037 18255305

[B4] Doll N Borger MA Hain J Bucerius J Walther T Gummert JF Mohr FW Minimal access aortic valve replacement: effects on morbidity and resource utilization Ann Thorac Surg 2002 74 4 S1318 S1322 10.1016/S0003-4975(02)03911-5 12400808

[B5] Bakir I Casselman FP Wellens F Jeanmart H De Geest R Degrieck I Van Praet F Vermeulen Y Vanermen H Minimally Invasive Versus Standard Approach Aortic Valve Replacement: A Study in 506 Patients Ann Thorac Surg 2006 81 5 1599 1604 10.1016/j.athoracsur.2005.12.011 16631641

[B6] Liu J Sidiropoulos A Konertz W Minimally invasive aortic valve replacement (AVR) compared to standard AVR Eur J Cardiothorac Surg 1999 16 Suppl 2 S80 S83 10613563

[B7] Aris A Cámara ML Montiel J Delgado LJ Galán J Litvan H Ministernotomy versus median sternotomy for aortic valve replacement: a prospective, randomized study Ann Thorac Surg 1999 67 6 1583 1587 discussion 1587–8 10.1016/S0003-4975(99)00362-8 10391259

[B8] Dogan S Dzemali O Wimmer-Greinecker G Derra P Doss M Khan MF Aybek T Kleine P Moritz A Minimally invasive versus conventional aortic valve replacement: a prospective randomized trial J Heart Valve Dis 2003 12 1 76 80 12578340

[B9] Murtuza B Pepper JR Stanbridge RD Jones C Rao C Darzi A Athanasiou T Minimal access aortic valve replacement: is it worth it? Ann Thorac Surg 2008 85 3 1121 1131 10.1016/j.athoracsur.2007.09.038 18291224

[B10] Brinkman WT Hoffman W Dewey TM Culica D Prince SL Herbert MA Mack MJ Ryan WH Aortic valve replacement surgery: comparison of outcomes in matched sternotomy and PORT ACCESS groups Ann Thorac Surg 2010 90 1 131 135 10.1016/j.athoracsur.2010.03.055 20609763

[B11] Detter C Deuse T Boehm DH Reichenspurner H Reichart B Midterm results and quality of life after minimally invasive vs. conventional aortic valve replacement Thorac Cardiovasc Surg 2002 50 6 337 341 10.1055/s-2002-35743 12457309

[B12] Machler HE Bergmann P Anelli-Monti M Dacar D Rehak P Knez I Salaymeh L Mahla E Rigler B Minimally invasive versus conventional aortic valve operations: a prospective study in 120 patients Ann Thorac Surg 1999 67 4 1001 1005 10.1016/S0003-4975(99)00072-7 10320242

[B13] Moustafa MA Abdelsamad AA Zakaria G Omarah MM Minimal vs median sternotomy for aortic valve replacement Asian Cardiovasc Thorac Ann 2007 15 6 472 475 18042770 10.1177/021849230701500605

